# Effect of *Morinda citrifolia* fruit powder on physiological and productive performance of *Cavia porcellus*

**DOI:** 10.3389/fvets.2023.1134138

**Published:** 2023-10-13

**Authors:** Daniel Marco Paredes-López, Rizal Alcides Robles-Huaynate, Xiomara Beteta-Blas, Uriel Aldava-Pardave

**Affiliations:** ^1^Department of Animal Science, Universidad Nacional Agraria de la Selva, Tingo María, Peru; ^2^Facultad de Ciencias, Universidad Nacional Agraria La Molina, Lima, Peru

**Keywords:** *Cavia porcellus*, growth promotor, gut health, *M. citrifolia*, wellbeing

## Abstract

The breeding of guinea pig is part of the pluriactivity for millions of farming families in rural areas from the Peruvian Andean and Amazonian regions and other South American Andean countries. Rearing these specie plays an important source of employment, income, and nutrition for millions of rural families on these countries. The search of natural products for enhancing animal wellbeing, health, and production and thereby of guinea pigs is being searched nowadays. The aim of this study was to determine the effect of the ripe fruit powder of *Morinda citrifolia* on the physiological and productive performance parameters of reared guinea pigs under humid tropical conditions and to find a new use of noni fruit and to improve the guinea pig as an agrifood product. For this purpose, forty-eight male Peru breed guinea pigs sixty days old, were used and distributed into four treatments with diets containing 0, 2, 4 and 8% of the noni ripe fruit powder, with four replicates and 3 guinea pigs each. Erythrocytes, hematocrit, hemoglobin profiles, hematological indices MCV (mean cell volume), MCHC (mean corpuscular hemoglobin concentration), MCH (mean corpuscular hemoglobin) and blood metabolites profiles: TP (total protein), ALB (albumin), GLO (globulin), TC (total cholesterol) were determined. The productive performance indices: DWG (daily weight gain), DCFI (daily concentrated feed intake), TFIFM (total feed intake of fresh matter) and TFIDM (total feed intake of dry matter), FRCFM (feed rate conversion for fresh mater) and FRCDM (feed rate conversion for dry matter) were evaluated. The guinea pigs were evaluated at 60, 75 and 90 days old. The interaction between noni fruit powder and the age of guinea pigs produced an increase in the erythrocyte, hematocrit, MCH and MCHC levels at 75 days old, (*p* < 0.05). The final weight and the daily weight gain increased, while the feed rate conversion for fresh and dry matter decreased, as the level of noni fruit powder in the diet increased until 4% (*p* < 0.05). Thus, the level of noni ripe fruit powder in the guinea pigs' diets had a positive effect on the erythrocyte, leucocytes, hematocrit, MCH, MCHC levels, the final weight, the daily weight gain, and the feed rate conversion of fresh and dry matter.

## Introduction

The rearing of guinea pig is part of the pluriactivity for millions of farming families in rural areas from the Peruvian Andean and Amazonian regions (1) and from other countries as Equator, Colombia, and Bolivia since ancient's civilizations. Rearing these specie plays an important source of employment, income, and nutrition for millions of rural families in those countries (2). However, the high level of adaptability has allowed to this specie for rearing technification, thus, improving the wellbeing, health and productive indices and as a result is being increased its commercial production nowadays (3).

The gastrointestinal problems during the different phases of production are the main causes of low productivity in the breeding of this species, and this is caused by various bacterial agents, them *Escherichia coli, Clostridium, Streptococcus*, and *Salmonella* (4–7).

The indiscriminate use of antibiotics, commonly used to reduce the colonization of these pathogens in the gastrointestinal tract, to control the development of gastrointestinal infection and promote the growth in livestock causes the development of antimicrobial resistance and the meat from these animals can contain residuals of these substances, thus, putting the public health and the environment at risk (8, 9).

This scenario brings us to research about new forms of controlling these pathogens through the use of plants containing active antimicrobial, antioxidant, and immunomodulation compounds; which, in an integral fashion, can act by moderating the microbiota, the structure, and the gastrointestinal function, as well as the immune system in livestock and poultry (10–13), and in this manner, promote the improvement of the wellbeing, health and productive performance of these animal species (14–19).

*Morinda citrifolia*, “noni,” is a plant native to southeast Asia and today is has expanded throughout the Caribbean, Central, North and South America (20, 21). It is known for its antimicrobial properties (22–24), as well as its immunomodulation activities (25–27) and antioxidant activity (25, 27–29). In the Amazon and high jungle regions of Peru, this plant is grown for traditional medicine and nutraceutical purposes (30) and produce abundant leaves and many fruits all year long, but this productive potential is not taken advantage locally. From this, stems our interest in researching new ways to take advantage of this plant, for the improvement of the wellbeing, health, and productive performance of guinea pigs. The purpose of this study was to evaluate the effect of different concentrations of noni fruit powder on the hematological, blood metabolites profiles and productive indices of guinea pigs during the fattening phase and to find a new use of noni fruit and to improve the guinea pig as an agrifood product.

## Materials and methods

### Variables considered in the study

Four levels of *M. citrifolia* powder and three age of guinea pigs were considered as independent variables, while the dependent variables are shown in Table 1.

**Table 1:** Independent and dependent variables considered in the study.

**Independent variables**	***Morinda citrifolia*: four levels (0, 2, 4, and 8%)**
	*Cavia porcellus* ag*e*: three ages (60, 75, and 90 days old)
Dependent variables	Hematological profiles
	Blood metabolites profiles
	Productive performances índices

### *Morinda citrifolia* fruit powder

The collection of the noni fruit was done in Tingo María, located at the geographic coordinates UTM 3981790 meters East and 8,973,000 meters North and 715 m.a.s.l. This collected material has been identified and registered as *M. citrifolia* with the code N° 63841 -HUT by the Herbarium Truxillense (HUT) from the Universidad Nacional de Trujillo. The botanical identification was based in its 20–34 cm hairless, opposite petiolate and glabrous leaves, white tubular in bunch flowers and fleshy multiple fruits. For the experiment was used 25 kilograms of whole fresh ripe fruit in a well conserved conditions, these were taken through a drying process at 60°C in a forced ventilation stove for 72 h to later be ground using a 1 mm diameter sieve in a Model 4 Thomas Willey brand grinder, USA, and stored in tightly sealed recipients, out of light. This procedure was adapted from Lal et al. (31).

### Experimental animals and nutrition

The localization of this study was at 09° 17′ 58″ south latitude and 76° 01′ 07″ west longitude, with an altitude of 660 m.a.s.l, an annual pluvial precipitation of 3,293 mm, an average annual temperature of 24.85°C and a relative humidity of 80% (32). Forty-eight, sixty-day old, male guinea pigs of the Peru breed, with an average weight of 499.00 ± 8.60 g were used; they were adapted to the diet 1 week prior to the beginning of the experimental evaluation. They were distributed into four treatments with four replicates for each one. These were reared in sixteen metal mesh cages of one meter area and half meter high located in a facility with manual controlled ventilation by polyethylene curtains. Guinea pigs were only handled from their cages for taking blood samples from the cephalic vein at 60, 75 and 90 days old. The ethical procedures applied during this experimental research were approved by the ethics committee from the Universidad Nacional Agraria de la Selva. The guinea pigs belonged to the guinea pigs production section from the Faculty of Zootechny, Universidad Nacional Agraria de la Selva, where the research was carried out. All the experimental animals were fed with a basal diet with the requirements for the fattening phase (33), made of concentrated feed 47% and forage 53%, (Tables 2–4) to which 0, 2, 4 and 8% of the noni fruit powder was added for each treatment considering a very marginal impact on guinea pigs performance because of its very low macronutrients content of this fruit (34, 35). The four treatments were done for 1 month period corresponding to 60–90 days old of guinea pigs. Hematological and blood metabolites profiles were evaluated at 60, 75 and 90 days old and the daily concentrated feed diet for each animal was 50 g and forage *ad libitum*.

**Table 2:** Composition of the concentrated feed in the basal diet for guinea pigs during the fattening phase.

**Ingredient**	**(%)**
Corn	41.26
Wheat bran	17.14
Soy cake	18.72
Alfalfa flour	15.00
Molasses	4.00
Palm oil	1.32
Calcium carbonate	1.25
Dicalcium phosphate	0.25
Salt	0.42
Vitamin mineral premix (Guinea pigs)	0.10
Micotoxin chelator	0.05
BHT	0.05
Choline chloride	0.10
Lysine	0.14
Methionine	0.17
Threonine	0.04
Total	100.00

**Table 3:** Nutritional composition of the basal diet for guinea pigs in the fattening phase.

**Components**	**Content**
Dry matter (%)	89.36
Total protein (%)	17.28
Digestible energy (kcal/kg)	2,978
Ether extract (%)	4.40
Total fiber (%)	7.18
Neutral detergent fiber (%)	21.49
Acid detergent fiber	9.78
Calcium (%)	0.99
Total phosphorus (%)	0.45
Sodium (%)	0.22
Total lysine (%)	0.96
Total methionine (%)	0.44
Total threonine (%)	0.70

**Table 4:** Proximal analysis and total energy of purple king grass as forage in the basal diet for guinea pigs in the fattening phase.

**Components**	**Content**
Dry matter (%)	25.00
Total protein (%)	1.98
Ether extract (%)	0.40
Total fiber (%)	9.43
Ash (%)	3.64
Nitrogen free extract (%)	9.55
Total energy (kcal/kg)	970.31

### Hematological and blood metabolites profile

Blood samples were obtained from the cephalic vein of experimental guinea pigs at 60, 75 and 90 days old; the erythrocytes counts were done using an automatic analyzer kontrolab BC H2 (Italy). The determination of the hematocrit was done using the microhematocrit method, at 11,000 rpm, for 3 mins (36) in a Tom's Kert Lab centrifuge (USA Science Tech Group). The determination of the hemoglobin was done using the cyanmethemoglobin method, for which the Drabkin's reagent was used. The indices mean cell volume (MCV), Mean corpuscular hemoglobin concentration (MCHC) and mean corpuscular hemoglobin (MCH) were calculated. For the biochemical analysis, the serum was obtained by centrifugation of the coagulated blood at 1,500 rpm for 5 mins. The total protein profile was determined using the Biuret colorimetric method, and for the albumin profile, the Bromocresol green method was used (37, 38), and the cholesterol profile was obtained using the enzymatic method; readings were done in an Auto Chemistry Analyzer-AS 830 spectrophotometers (Italy) at 515 and 530 nm using specific kits (QAC-Spain).

### Productive performance

To determine the effect of the different levels of *Morinda citrifolia* fruit powder on the guinea pigs productive performance, daily consumed and left forage and concentrated feed given to all experimental guinea pigs were recorded during the 30 days of experiment. Body weight of all experimental guinea pigs were recorded at 60, 75, and 90 days old. Using this data and adapting conventional productive performance ratios for animal production (39) the initial weight, final weight, daily weight gain, concentrated daily feed intake, concentrated total feed intake total feed intake of fresh matter, total feed intake of dry matter, fresh matter feed rate conversion, and dry matter feed rate conversion were recorded. In the experiment was consider the concentrated feed and forage as different source of feed and fresh and dry mater as different condition of the feed to evaluate the possible response of the guinea pigs in different way to each source and condition of the feed in presence of the noni rip fruit powder. Each of these productive performance were calculated by mean of the following ratios:


$$\begin{array}{l} DWG:\text{Dailyweightgain}(\text{g})\text{ =}\\ \frac{\text{Final weight}\left(\text{g}\right)\text{per guinea pig}}{\text{Number of evaluated days}} \end{array}$$

$$\begin{array}{l} CFDI:\text{Concentrated feed daily intake }\left(\text{g}\right)\text{=}\\ \;\frac{\text{Feed offered }(\text{g})\text{-feed refused}(\text{g})}{\text{Number of evaluated days}} \end{array}$$

$$\begin{array}{l} CFTI:\text{ Concentrated feed total intake}\left(\text{g}\right)\text{=}\\ \text{ Feed offered }(\text{g})-\text{feed refused}(\text{g}) \end{array}$$

$$\begin{array}{l} \;TFIFM:\text{ Total feed intake of fresh matter}\left(\text{g}\right)\text{=}\\ \;(\text{Feed offered }\left(\text{g}\right)\left(\text{concentrated + forage}\right)-\\ \text{feed refused}\left(\text{g}\right)\;\left(\text{concentrated + forage}\right))\text{ as offered} \end{array}$$

$$\begin{array}{l} \;TFIDM:\text{Totalfeedintakeofdrymatter}(\text{g})\text{=}\\ \;(\text{Feed offered }\left(\text{g}\right)\left(\text{concentrated + forage}\right)-\\ \text{feedrefused}(\text{g})(\text{concentrated + forage}))\text{ 100}\%\text{ dry matter} \end{array}$$

$$\begin{array}{l} \;FMFR\text{C}:\text{ Fresh matter feed rate conversion }\left(\frac{\text{g}}{\text{g}}\right)\text{=}\\ \frac{\left(\text{Total Feed offered }(\text{g})\;\left(\text{concentrated + forage}\right)\right)\text{ as offered}}{\text{Final weight x number of guinea pigs}} \end{array}$$

$$\begin{array}{l} \;DMFRC:\text{ Dry matter feed rate conversion }\left(\frac{\text{g}}{\text{g}}\right)\text{=}\\ \frac{\left(\text{daily Feed offered }\left(\text{g}\right)\left(\text{concentrated + forage}\right)\right)\text{ 100}\%\text{ dry matter}}{\text{Final weight x number of guinea pigs}} \end{array}$$

### Statistical analysis

For the evaluation of the effect of the *M. citrifolia* powder on the blood metabolites and hematological profiles, a general factorial design (40) with two factors, 4 levels of noni fruit powder and 3 ages of guinea pigs to show the effects of the individual factors or their interactions on the response were used.

The variance analyses were done with Infostat statistical software (41) and the averages were compared using the Student-Newman-Keuls test (5%). For determining the linear or quadratic effect of *M. citrifolia* fruit powder levels on the productive parameters of guinea pigs a linear regression analysis of data was carried out.

## Results

### Hematological profiles

**Figure 1:**
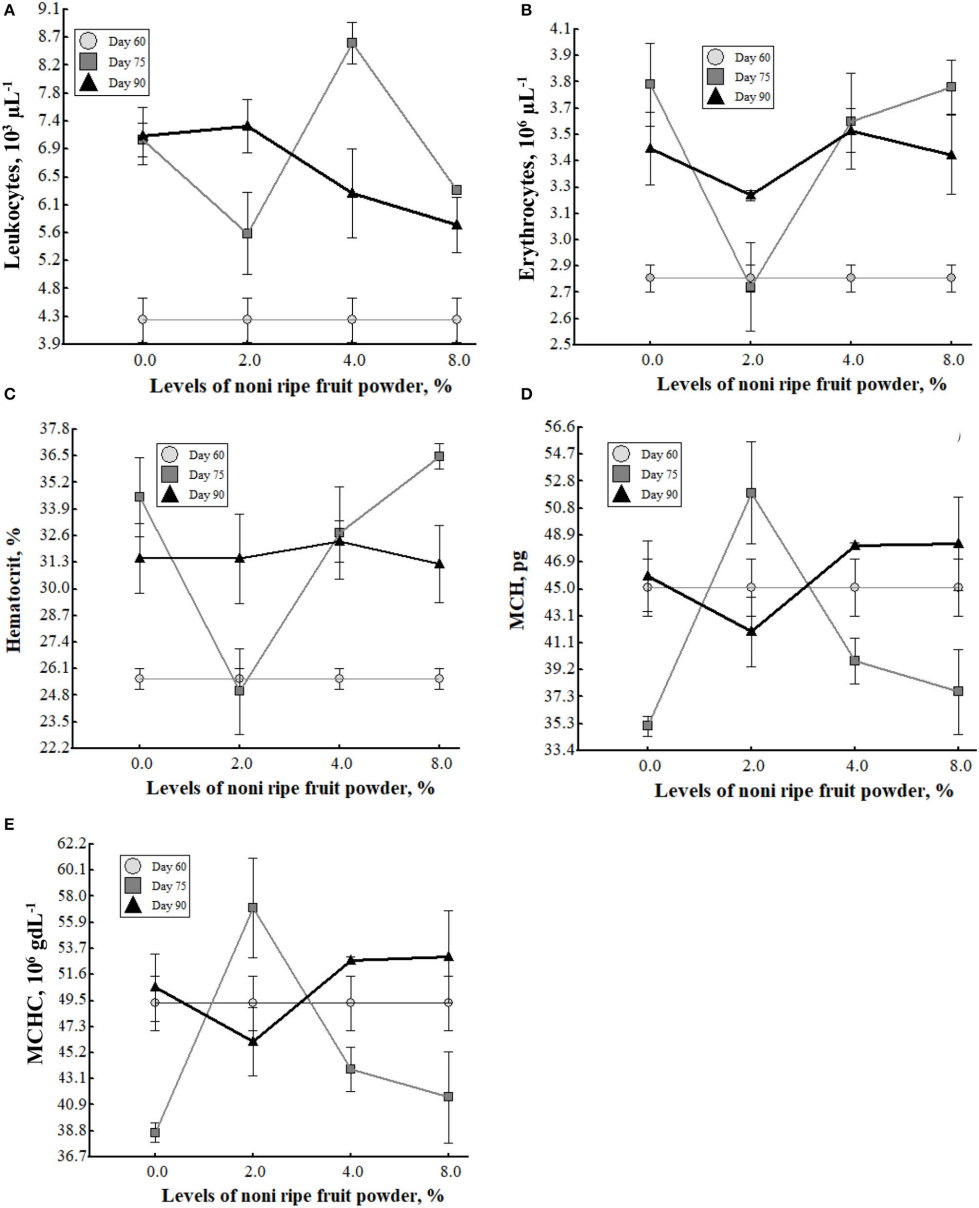
Hematological profiles under the interaction between noni ripe fruit powder levels and guinea pig ages on Leukocyte **(A)**, Erythrocyte **(B)**, Hematocrit **(C)**, MCH **(D)** and MCHC **(E)** values.

The total number of leucocytes, erythrocytes, hematocrit, MCH and MCHC increased (*p* < 0.05) by interaction between the level of noni fruit powder (NFP) and the guinea pigs age (GPA), being at 75 days old and 4% of noni fruit powder in the diet, the highest level (Tables 5, 6 and Figure 1), however, hemoglobin and mean cell volume did no varied (*p* > 0.05) (Tables 5, 7).

**Table 5:** ANOVA of hematological profiles of guinea pigs at 60, 75 and 90 days old fed with different levels of noni ripe fruit powder.

**Source of variation**	**ANOVA F (*****p*** **values)**						
	WBC^*^(10^3^ μ**L**^−1^**)**	RBC^*^ (10^6^ μ**L**^−1^**)**	**HCT** ^*^ **(%)**	**HBG (gdL** ^−1^ **)**	**MCV (fL)**	**MCH (pg)**	**MCHC (gdL** ^−1^ **)**
Noni level (NL)	0.2450	0.0037	0.0449	0.0177	0.9443	0.2654	0.2910
Guinea pigs age (GPA)	0.0001	0.0001	0.0001	0.0001	0.0001	0.0257	0.0372
NL ^*^ GPA	0.0598	0.0217	0.0166	0.4392	0.9924	0.0034	0.0048
CV (%)	20.3798	9.5845	11.2746	6.7818	0.0561	11.8065	12.0732
R^2^ adjusted (%)	52.8248	60.0143	53.3113	63.3067	90.8339	35.1910	32.8709

Comparisons were done by the student-Newman-Keuls (SNK) test. WBC, leukocytes; RBC, erythrocytes; HCT, Hematocrit; HBG, Hemoglobin; MCV, mean corpuscular volume; MCH, mean corpuscular hemoglobin; MCHC, mean corpuscular hemoglobin concentration. ^*^Data were transformed by log (10).

**Table 6:** Hematological profiles under the interaction effect of guinea pigs age ^ABC^ and noni ripe fruit powder level in the diet^abc^.

**Variable**	**Age of guinea pigs (days)**	**SEM**	**Noni ripe fruit powder (%)**			
			**0%**	**2%**	**4%**	**8%**
WBC (10^3^μL–^1^)	60	0.60	4.27^B^	4.27^B^	4.27^C^	4.27^B^
	75		7.08^bA^	5.63^cB^	8.60^aA^	6.30^bcA^
	90		7.15^A^	7.30^A^	6.25^B^	5.76^A^
RBC (10^6^μL–^1^)	60	0.15	2.80^B^	2.8	2.80^B^	2.80^B^
	75		3.80a^A^	2.75^b^	3.61a^A^	3.78^aA^
	90		3.47^A^	3.22	3.56^A^	3.43^A^
HCT (%)	60	1.68	25.63^B^	25.63	25.63^B^	25.63^C^
	75		34.5a ^A^	25.00^b^	32.75^aA^	36.50^aA^
	90		31.50^A^	31.5	32.33^A^	31.25^B^
MCH (pg)	60	2.60	45.11A	45.11	45.11^A^	45.11
	75		35.15bB	51.92a	39.81^bB^	37.60^b^
	90		45.93A	41.94	48.11^A^	48.26
MCHC (gdL^−1^)	60	2.92	49.25A	49.25	49.25^A^	49.25
	75		38.66bB	57.03^a^	43.82^bB^	41.56^b^
	90		50.53A	46.10	52.74^A^	53.03

ABC: Different capital letters within the same column indicate statistical differences for guinea pig age. abc: Different lowercase letters within the same line indicate statistical differences for noni fruit powder level within the same age (SNK 5%). WBC, leukocytes; RBC, erythrocytes; HCT, Hematocrit; MCH, mean corpuscular hemoglobin; MCHC, mean corpuscular hemoglobin concentration.

**Table 7:** Hematological profiles of guinea pigs at 60, 75 and 90 days old fed with different levels of noni ripe fruit powder.

**Factors**	**WBC (10^3^μL^−1^)**	**RBC** **(10^6^μL^−1^)**	**HCT (%)**	**HBG (gdL^−1^)**	**MCV (fL)**	**MCH (pg)**	**MCHC (gdL^−1^)**
**Age of Guinea pigs (days)**							
SEM	0.30	0.08	0.84	0.24	0.01	1.30	1.46
60	4.27^b^	2.80^b^	25.63^b^	12.52^c^	72.72^a^	45.11^a^	49.25^ab^
75	6.90^a^	3.48^a^	32.19^a^	14.20^b^	72.46^b^	41.12^b^	45.27^b^
90	6.62^a^	3.42^a^	31.65^a^	15.42^a^	72.45^b^	46.06^a^	50.60^a^
**Noni ripe fruit powder (%)**							
SEM	0.35	0.09	0.97	0.28	0.01	1.50	1.69
0%	6.17^a^	3.35^a^	30.54^ab^	14.23^ab^	72.54^a^	42.07^a^	46.15^a^
2%	5.73^a^	2.92^b^	27.38^b^	13.36^b^	72.54^a^	46.32^a^	50.79^a^
4%	6.37^a^	3.32^a^	30.24^ab^	13.97^ab^	72.54^a^	44.34^a^	48.60^a^
8%	5.44^a^	3.34^a^	31.13^a^	14.64^a^	72.55^a^	43.66^a^	47.95^a^

abc: Different letters within the same column indicate statistical differences (SNK 5%). WBC, leukocytes; RBC, erythrocytes; HCT, Hematocrit; HBG, Hemoglobin; MCV, mean corpuscular volume; MCH, mean corpuscular hemoglobin; MCHC, mean corpuscular hemoglobin concentration.

### Blood metabolites profile

Results of blood metabolites profiles are presented in Tables 8, 9 and total protein, albumin, globulins and total cholesterol profiles of the guinea pigs did not vary (*p* > 0.05) under the effect of the three levels of noni fruit powder included in the diets (Tables 8, 9). The protein and albumin profiles in the blood serum decreased as the age of guinea pigs increased but the globulin increased from 60 to 75 days old of the guinea pigs (*p* < 0.05) (Tables 8, 9).

**Table 8:** ANOVA of blood metabolites profiles of guinea pigs at 60, 75, and 90 days' old fed with different levels of noni ripe fruit powder.

**Sources of variation**	**ANOVA F (** ***p*** **-values)**			
	**TP (gdL** ^−1^ **)**	**ALB** **(gdL**^−1^**)**	**GLO (gdL** ^−1^ **)**	**TC** **(gdL**^−1^**)**
Noni level (NL)	0.3412	0.1201	0.4859	0.4267
Age of Guinea pigs (AGP)	0.5554	0.0001	0.0158	0.1728
NL ^*^ AGP	0.3825	0.3910	0.6407	0.3036
CV (%)	6.2475	8.7285	40.3689	14.3411
R^2^ adjusted (%)	71.6053	61.9147	9.8016	6.0970

Comparisons were done by the student-Newman-Keuls (SNK) test. TP, total protein; ALB, albumin; GLO, globulin; TC, total cholesterol.

**Table 9:** Blood metabolites profiles of guinea pigs at 60-, 75-, and 90-days' old fed with different levels of noni ripe fruit powder.

**Factors**	**TP (gdL^−1^)**	**ALB(gdL^−1^)**	**GLO (gdL^−1^)**	**TC (gdL^−1^)**
**Age of** ***Guinea pigs, days***				
SEM	0.08	0.08	0.11	1.06
60	5.27	4.35^a^	0.87^b^	30.93
75	5.13	3.86^b^	1.36^a^	29.66
90	7.39	3.32^c^	1.12^ab^	28.06
**Noni levels, (%)**				
SEM	0.09	0.10	0.13	1.22
0%	4.99	3.87	1.11	29.06
2%	4.76	4.01	1.13	28.69
4%	4.80	3.81	0.97	29.13
8%	4.95	3.68	1.26	31.31

abc: Different letters within the same column indicate statistical differences (SNK 5%).TP: total protein.

### Productive performance

**Figure 2:**
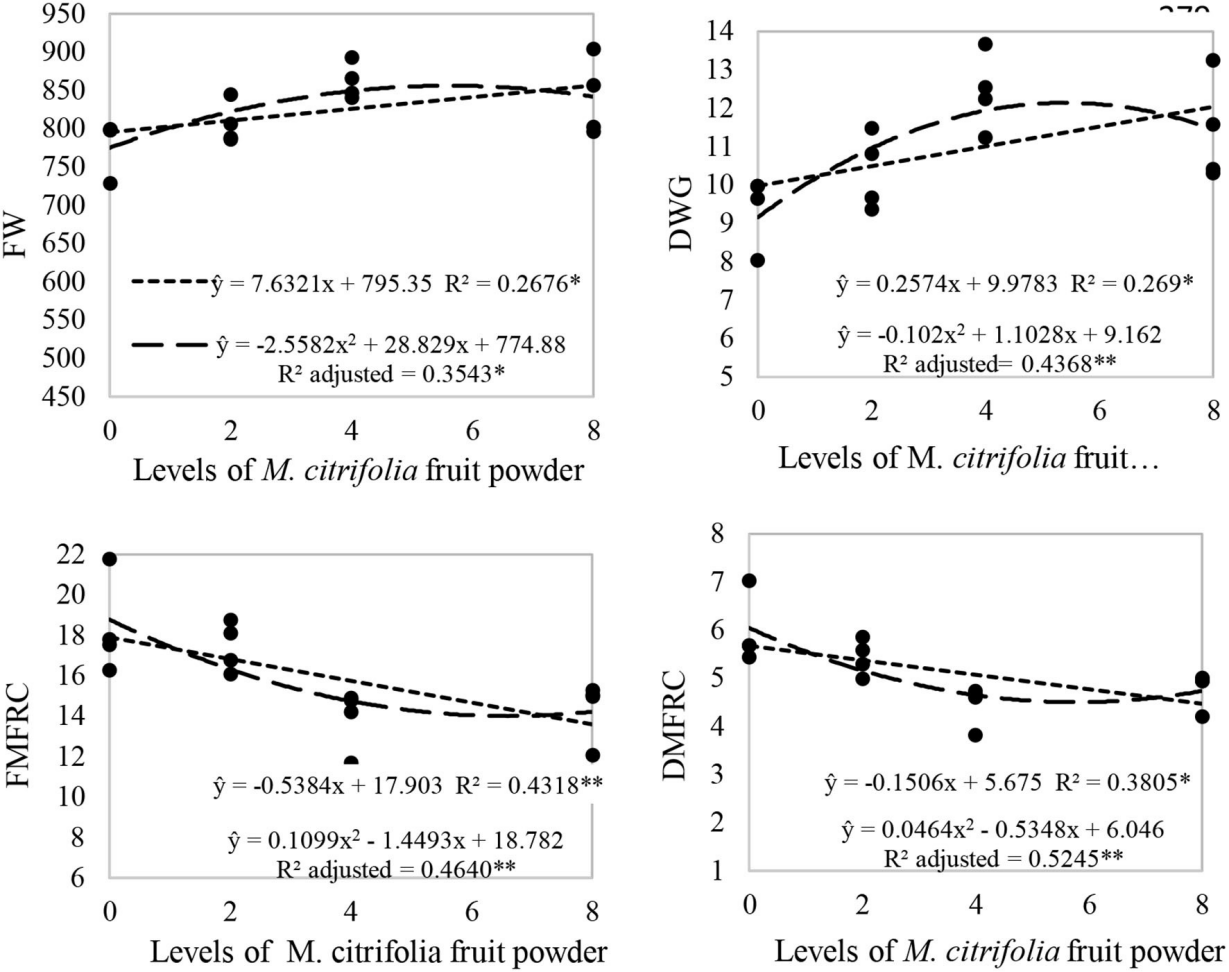
Linear regression analysis of the effect of noni ripe fruit powder on four productive indices of guinea pigs. *, ** Statistical difference (*p* < 0.05 and *p* < 0.01 respectively). FW, final weight; DWG, daily weight gain; FMFRC, fresh matter feed rate conversion; DMFRC, dry matter feed rate conversion.

Results of production performance indices are in Table 10 and Figure 2. Final weight and daily weight gain increased as the inclusion of noni powder in the diets increased up to 4%; the FW increased from 781.00 g to 862.00 g and the DGW increased from 9.40 g to 12.42 g (*p* < 0.05). In parallel to these two indices, the fresh matter feed rate conversion and dry matter feed rate conversion were reduced as the inclusion of noni fruit powder in the diets increased up to 4% (*p* < 0.05); decreasing from 18.37 ± 2.37 to 13.91 ± 1.50 kg of fresh matter per kilogram of meat and from 5.95 ± 0.72 to 4.45 ± 0.43 kg of dry matter per kilogram of meat (Table 10, Figure 2).

**Table 10:** Productive performance of fattening guinea pigs fed different levels of noni ripe fruit powder in the diet.

**Performance indices**	**Levels of noni fruit powder (%)**				***p*-value**	**SEM**
	**0%**	**2%**	**4%**	**8%**		
IW (g)	499.00	496.00	489.00	499.00	0.5172	2.31
FW (g)	781.00^b^	806.00^ab^	862.00^a^	840.00^a^	0.0344	17.82
DWG (g)	9.40^c^	10.33^cb^	12.42^a^	11.38^ab^	0.0109	0.65
CDAC (g)	31.18	29.33	30.30	31.61	0.2459	0.50
CDFI (g)	140.00^ab^	150.00^a^	142.00^ab^	130.00^b^	0.0794	4.00
TDFIFM (g)	171.18	179.33	172.30	161.61	0.1713	3.53
TDFIDM (g)	55.43	55.77	54.99	53.88	0.7735	0.41
FMFRC (g/g)	18.37^b^	17.44^b^	13.91^a^	14.36^a^	0.0066	1.11
DMFRC(g/g)	5.95^c^	5.43^bc^	4.45^a^	4.77^ab^	0.0049	0.34

abc: Different letters within the same column indicate statistical differences (SNK 5%). IW, initial weight; FW, final weight; DWG, daily weight gain; CFDI, Concentrated feed daily intake; CFTI, Concentrated feed total intake; TFIFM, total feed intake of fresh matter; TFIDM, total feed intake of dry matter; FMFRC, fresh matter feed rate conversion; DMFRC, dry matter feed rate conversion.

## Discussion

### Hematological profiles

Blood is the main tissue that produce cells for transporting oxygen which is the power in cells metabolism (42). The increase of total number of leucocytes, erythrocytes, hematocrit, MCH and MCHC by interaction in the present research have been also obtained in an experiment where the administration of noni extract for mice that were intoxicated with methotrexate, the leucocytes and erythrocytes reduced by the toxic effect of this substance increased and re-established by the effect of the noni extract (43). Similar results were also obtained in leucocytes response in rats clinically healthy, in which the administration of 5 mg/kg (live weight) of noni extract increased the leukocyte population, when compared to those that did not receive treatment, but no effect on the hemoglobin and erythrocyte profiles were shown (43, 44). The increase in total number of leucocytes, erythrocytes, hematocrit, MCH and MCHC in the present study may be due to the guinea pigs response to the phytochemical compounds such as triterpenes, steroids, amino acids, alkaloids, and flavonoids, which confer the antioxidant and immunomodulation effects of the fruit powder of *M. citrifolia* included in the diet (25, 28, 29, 44–48) and the negative feedback mechanisms to increase the number of leucocytes and erythrocytes as response to an increase of a metabolic demand from 60 to 75 days old of the guinea pigs as an interaction.

Furthermore, all the evaluated hematological profiles increased in relation to the guinea pigs age (p <0.05) (Tables 5, 7), these results have their origin in the physiological mechanisms, the negative feedback produced by the increase in metabolic demand, that pushes blood cell profiles to increase as the specimen matures (42).

The hematological profiles in the present study varied within the normal ranges or average intervals for each profile in the specie (49–52).

### Blood metabolites profile

No changes in total protein profile in our research have been also obtained in healthy mice that were treated with 5mg/kg of noni extract/live weight, in which, the protein level in the serum increased in comparison with the control group (43). Similar results for those obtained in the present study for albumin and globulin profiles which did not change under the effect of noni fruit powder have been also obtained in plasma of healthy mice when treated with different concentrations of noni juice (44). The total cholesterol profile of our study has been also obtained in mice treated with noni extract in which this metabolite did not vary in comparison to the group with no treatment (43).

The reduction in protein and albumin profiles and increase in globulin in the blood serum as the age of guinea pigs increased from 60 to 75 days old of the guinea pigs (*p* < 0.05) contrast the levels of serum protein produced by the physiological mechanisms, in which the total protein levels in serum, as well as the albumin and globulin levels increase as the adult stage is reached in animal species (37, 42).

However, decrease in the total protein and albumin in the blood as the age of guinea pigs increased in the current study could be related to an increase in the consumption of the feed containing greater levels of noni powder, and some bioactive compounds of this fruit enhance the storage or retention of protein in the guinea pigs' tissues; as for those obtained in Nile tilapia (53). It may be one of the mechanisms for improving the final weight and daily weight gain in the performance indices in the guinea pigs obtained in the present study. At the same time, it may be related with a nephroprotective effect of *M. citrifolia* leaves powder by maintaining tubular and glomerular epithelial cells, decreasing serum protein but between normal ranges (54).

Different results of protein and albumin profiles in guinea pigs were found in a study using different levels of *Erythrina sp*. leaves powder with the purpose of finding an alternative source of protein for guinea pigs. In this study the protein and albumin profiles in the serum increased as the level of *Erythrina sp*. powder in the diets increased, notwithstanding, a gradual decrease in the live weight, carcass weight, and carcass yield were obtained each time that a greater level of *Erythrina sp*. leaves powder was added in the diet (55).

The blood metabolites profiles in the present study varied within the normal ranges or average intervals for each profile in the specie (49, 50, 52).

### Productive performance

Eventhough the indirect assessment of the productive performance by feed intake and weight gain is not accurate enough, those are worldwide considered as the main primary assessments from which are derived the main ratios for evaluating productivity in animal production. Increase in weight gain and reduction of the feed rate conversion are the main indices when a productive performance is evaluated (39). These results have been obtained by the addition of ripe fruit powder in the diet of guinea pigs in the present study. Similar results have also been reported in the Nile tilapia for which the addition of noni extract in the diets, increased the specific growth index and the daily length, and decreased the feed rate conversion (53); in another study in growing cattle, an increase in the daily weight gain was obtained as the addition of noni fruit pulp in the diets increased (56).

One explanation of the improvement in the productive indices in guinea pigs may be associated with some effect of growth promotion from the noni fruit powder since it contains antimicrobial compounds and endophytic bacteria with potent inhibitory activity on the intestinal microbiota, in such a way that they regulate the intestinal microbiome, and in this manner may strengthen the gut health of the guinea pigs (23, 24, 57–59).

This improvement could be also associated with an increase in the profiles of the number of erythrocytes, hematocrit, MCH and MCHC, due to their antioxidant properties (44, 45, 47), by which *M. citrifolia*, has been found to be protective against tissues metabolic stress instead of any toxic actions (25, 28), thus improving the oxygenation of tissues and their metabolic activity (42). At the same time, some bioactive compounds of this fruit do protect the liver from injuries by inhibiting inflammation (48) and by means of this mechanism may enhances absorption process and increase the storage or retention of protein in the guinea pigs' tissues; as for those obtained in Nile tilapia (53).

Nonetheless, the results in our study differ from those obtained in a study of the powder from ripe and unripe fruit from this plant on the productive performance of chickens, which did not influence their productive indices, nor the digestibility of the nutrients in chickens' diets (31); as well as in a study with mice, where the condition of their weight did not vary when given noni juice at different concentrations (44). These contrasting results may be explained because guinea pigs are herbivorous animals and their digestive physiology are specialized to degrade vegetable tissues more efficiently than granivorous animals as chickens and to extract phytochemical compounds which are beneficial for enhancing mechanisms of growth performance (60).

Feed intake was not impacted by supplementation of the noni ripe fruit powder across all treatments in this study. Previous study with noni extract in chickens agree with this finding (31) and in pigs fed with Origanum have been reported a dose related detrimental effect on palatability and therefore reducing feed intake (61).

Very few studies that involve studying the benefits of *M. citrifolia* in animal species to improve their wellbeing, health, and production are reported in the literature; thus, the present study is one of these few on this topic.

## Conclusions

This study explored the effects of noni rip fruit powder on production performance indices, hematological and blood metabolites profiles of guinea pigs in the fattening phase. The final weight, daily weight gain, and feed rate conversion for fresh and dry matter improved in the guinea pigs in the fattening phase fed diets containing 4% of rip fruit powder. At the same time, an increase in the leucocytes, erythrocytes, hematocrit MCH and MCHC profiles at 75 days old and 4% of noni ripe fruit powder in the diet produced by the interaction between these two variables was obtained. These results suggest that noni rip fruit powder may be used as a no conventional feed source for improving productive performance indices in traditional and commercial rearing systems of guinea pigs at 4% of the diet and to feed from 60 to 75 days old. Notwithstanding of that, the mechanism of action of Noni in guinea pigs remains to be studied.

## Data availability statement

The original contributions presented in the study are included in the article/supplementary material, further inquiries can be directed to the corresponding author.

## Ethics statement

The ethical procedures applied during this experimental research were approved by the Ethics Committee from the Universidad Nacional Agraria de la Selva.

## Author contributions

DP-L contributed to the conception and design of the study, reviewed the scientific literature, participated in the laboratory experiments, analyzed the data, and wrote the manuscript. XB-B contributed to the field experiments and laboratory work during guinea pigs rearing, analyzed the data, reviewed the scientific literature, wrote the manuscript, and sought funds to finance the study. RR-H contributed to the design and planning of the study, the review of the scientific literature, supervised the data analysis, and corrected the manuscript. UA-P supervised the field and laboratory work and analyzed the data, revised the manuscript. All authors contributed to the article and approved the submitted version.
